# Regorafenib in Japanese patients with solid tumors: phase I study of safety, efficacy, and pharmacokinetics

**DOI:** 10.1007/s10637-013-9953-8

**Published:** 2013-04-04

**Authors:** Yu Sunakawa, Junji Furuse, Takuji Okusaka, Masafumi Ikeda, Fumio Nagashima, Hideki Ueno, Shuichi Mitsunaga, Kensei Hashizume, Yuichiro Ito, Yasutsuna Sasaki

**Affiliations:** 1 International Medical Center-Comprehensive Cancer Center, Saitama Medical University, 1397-1 Yamane, Hidaka, Saitama 350-1298 Japan; 2 Department of Medical Oncology, Kyorin University, School of Medicine, Tokyo, Japan; 3 Hepatobiliary and Pancreatic Oncology Division, National Cancer Center Hospital, Tokyo, Japan; 4 Division of Hepatobiliary and Pancreatic Oncology, National Cancer Center Hospital East, Chiba, Japan; 5 Global Drug Discovery, Clinical Pharmacology, Bayer Yakuhin, Ltd., Osaka, Japan; 6 Product Development Department, Bayer Yakuhin, Ltd., Osaka, Japan; 7 Division of Medical Oncology, Department of Medicine, Showa University School of Medicine, Tokyo, Japan

**Keywords:** Regorafenib, Multikinase inhibitor, Solid tumors, Japanese patients

## Abstract

The safety, pharmacokinetics, and antitumor activity of the multikinase inhibitor regorafenib in Japanese patients was assessed in this multicenter, single-arm, phase I trial. Fifteen patients with treatment-refractory advanced solid tumors received regorafenib 160 mg once daily for the first 3 weeks of each 4-week cycle until disease progression, unacceptable toxicity, or investigator or patient decision to stop. The median duration of treatment was 2.1 months (range, 0.9–20.1 months). At data cutoff, one patient was still receiving regorafenib in cycle 21. Reasons for treatment discontinuation were disease progression (*n* = 12) and adverse events (liver enzyme elevation *n* = 1; anemia *n* = 1). Adverse events necessitated dose reduction in six patients, interruption of daily treatment in seven patients, and cycle delay in four patients. All patients experienced at least one drug-related adverse event, particularly gastrointestinal (87 %), dermatologic (73 %), or hematologic (67 %) events. There was no significant change in time to maximum concentration or terminal half-life of regorafenib and its active metabolites M2 and M5 between single dosing and 21-day continuous dosing. The area under the concentration–time curve was 2.1-fold higher for regorafenib, 5.2-fold higher for M2, and 37.3-fold higher for M5, and the maximum concentration was 2.0-fold, 4.8-fold, and 36.0-fold higher, respectively, after continuous dosing than after single dosing. One patient had a partial response (duration 10.5 months) and seven patients had stable disease. This study indicates that regorafenib 160 mg orally once daily (21 days on/7 days off treatment) can be given to Japanese patients who have solid tumors, without undue toxicity.

## Introduction

Tumor growth and disease progression involve many processes mediated by receptor tyrosine kinases, including angiogenesis [1], oncogenesis [2], and stromal interactions [3]. Blockade of multiple kinases involved in these processes is likely to achieve optimal inhibition of tumor growth and progression [4].

Regorafenib (BAY 73-4506; Bayer Pharma AG, Berlin, Germany) is a small-molecule inhibitor of multiple protein kinases, including those involved in angiogenesis (vascular endothelial growth factor receptors 1, 2, and 3, tyrosine kinase with immunoglobulin and epidermal growth factor homology domain 2), oncogenesis (KIT, RET, RAF-1, BRAF), and the tumor microenvironment (platelet-derived growth factor receptor-β, fibroblast growth factor receptor) [4].

Regorafenib has shown a broad spectrum of antitumor activity in preclinical xenograft tumor models [4]. Clinical efficacy has been observed in trials conducted in Europe and North America, including two global phase III studies involving patients with colorectal cancer (CRC) or gastrointestinal stromal tumors (GIST) [5–10].

The present study (NCT00960258) was conducted to define the pharmacokinetics and to assess the safety of regorafenib in Japanese patients with advanced solid tumors and to evaluate whether the dose and schedule selected for use in the global phase III trials (based on the European phase I dose-escalation trial [5]) would be appropriate for Japanese patients, before Japanese centers began participation in these studies.

## Materials and methods

This study was designed as a multicenter, single-arm, open-label, phase I, feasibility trial to assess the safety, pharmacokinetics, and preliminary efficacy of regorafenib, using the dose and schedule selected for the global phase III trials, in Japanese patients with treatment-refractory solid tumors. The study was conducted in accordance with the principles of the Declaration of Helsinki and Good Clinical Practice, as well as all local legal and regulatory requirements. The protocol was approved by each participating site’s institutional review board or independent ethics committee before the start of the study and before implementation of any amendments. Patients provided written informed consent to participate before enrollment into the study and again after Cycle 1 before proceeding to Cycle 2. The study was conducted at four study centers in Japan; at each center, the principal investigator was responsible for the study. The primary objectives of the study were to define the pharmacokinetics and to evaluate the safety of regorafenib in Japanese patients with advanced solid tumors. Secondary objectives included tumor response.

### Patient selection

Study eligibility criteria included histologically or cytologically confirmed solid tumors that were refractory to standard treatment or for which no standard treatment was available. Patients had to be aged 18 years or older and have Eastern Cooperative Oncology Group (ECOG) performance status 0 or 1 and an estimated life expectancy of at least 3 months. They also had to have adequate bone marrow, liver, and renal function in the 7 days before the start of treatment (i.e., hemoglobin ≥9.0 g/dl, absolute neutrophil count ≥1,500/mm^3^, platelet count ≥100,000/mm^3^, total bilirubin ≤1.5 × upper limit of normal [ULN], alanine aminotransferase [ALT] and aspartate aminotransferase [AST] ≤2.5 × ULN, serum creatinine ≤1.5 × ULN). The main exclusion criteria were as follows: previous or ongoing illness such as heart disease, uncontrolled hypertension, chronic hepatitis, coagulation or thrombotic disorder, renal impairment, or metastatic brain or meningeal tumor; surgery within 4 weeks before the first dose of regorafenib; inability to swallow oral medications; pregnancy or breast feeding; known or suspected allergy to the investigational agent; and use of anticancer chemotherapy, immunotherapy, bevacizumab, or any drugs that target vascular endothelial growth factor or its receptors within 4 weeks before the first dose or during the study.

### Dosage and schedule

Regorafenib was administered orally once daily after breakfast. The fixed starting dose was 160 mg orally once daily—this dose was selected in the European phase I dose-escalation trial on the basis of optimal balance of efficacy and tolerability. Predefined dose reductions (to 120 or 60 mg once daily) or interruptions (up to 28 days) were allowed if the patient experienced clinically significant grade 3/4 hematologic or other toxicities (e.g. grade 2 symptomatic/persistent hypertension or hand–foot skin reaction with no improvement after 7 days, or occurring for the second or third time) that were deemed to be related to the study medication. Treatment was discontinued if the patient required a dose reduction to less than 60 mg once daily or treatment interruption for more than 28 days. Dose re-escalation could be considered if the toxicity was not hematologic and had resolved to baseline; dose re-escalation was not permitted for reductions due to hematologic toxicities. Treatment was discontinued on progression or recurrence of the underlying cancer.

Patients were initially treated with one dose of regorafenib followed by 6 days off treatment (Cycle 0). They then started Cycle 1, during which they were given regorafenib once daily for 21 days, followed by 7 days off treatment. Patients who completed Cycle 1 with no unacceptable toxicities could continue with further cycles of regorafenib treatment (21 days on, 7 days off treatment), as long as they again provided written informed consent before Cycle 2, until they either progressed or met any of the discontinuation criteria, including treatment interruption for more than 28 days or need for further dose reduction below 60 mg/day to manage toxicity; pregnancy or intercurrent illness; patient, investigator, or sponsor’s request; non-compliance with the requirements of the study; or loss to follow-up.

### Patient evaluation

All patients who received at least one dose of regorafenib were eligible for safety analyses and were included in the efficacy analyses if they also had post-baseline efficacy data. For the safety analysis, physical examination, vital signs, use of concomitant medications, and laboratory test data were reported, and patients were routinely monitored for adverse events, which were recorded with severity and relationship to study medication according to the National Cancer Institute Common Terminology Criteria for Adverse Events version 3.0. Tumor response was assessed every 8 weeks for the first 6 cycles and every 12 weeks after Cycle 6 by Response Evaluation Criteria in Solid Tumors version 1.0, using the same imaging techniques and methods as at baseline. The response rate was defined as the proportion of patients whose best response was a confirmed partial or complete response.

### Definition of unacceptable toxicity and tolerability

Unacceptable toxicity was defined as any of the following events occurring during Cycle 1 and considered to be related to study drug: grade 4 neutropenia (for ≥7 days); febrile neutropenia with grade 4 neutropenia and fever; grade 4 thrombocytopenia; grade 3 or 4 nonhematologic toxicities (excluding events considered to be tolerable by the investigator). If two of the first three or three of the first six patients treated with regorafenib experienced unacceptable toxicities during Cycle 1, the safety data were reviewed by the Data Monitoring Committee, which considered whether protocol amendments were needed to address any safety concerns before subsequent patients were treated.

### Pharmacokinetics

Patients who provided at least one evaluable pharmacokinetic sample were eligible for pharmacokinetic analyses. Patients who did not receive multiple dosing of regorafenib 160 mg once daily for 21 days in Cycle 1 were not included in the analyses.

Blood samples (2 mL aliquots) for the determination of plasma concentration of regorafenib and its *N*-oxide metabolite M2 (BAY 75-7495) and *N*-oxide/*N*-desmethyl metabolite M5 (BAY 81-8752) were collected before the first dose (0 h) and 0.5, 1, 2, 3, 4, 6, 8, 12, 24, 36, 48, 72, and 96 h after dosing on Day 1 of Cycle 0 (single-dosing period at 160 mg), and before the first dose (0 h) and 0.5, 1, 2, 3, 4, 6, 8, 12, 24, 36, 48, 72, 96, and 168 h after dosing on Day 21 of Cycle 1 (multiple-dosing period at 160 mg).

Plasma concentrations of regorafenib and its metabolites were determined by fully validated liquid chromatography–tandem mass spectrometry after protein precipitation with acetonitrile/ammonium acetate buffer containing the internal standards [^2^H_3_^15^N] BAY 73-4506 (for regorafenib), [^2^H_3_^15^N] BAY 75-7495 (for the M2 metabolite), and [^2^H_3_^13^C^15^N] BAY 81-8752 (for the M5 metabolite). The pharmacokinetic parameters of regorafenib, M2, and M5 were calculated using the model-independent (noncompartment) method and the PC program WinNonlin (Version 4.1a; Pharsight Corporation).

### Sample size calculation and statistical analysis

No formal statistical sample size estimation was performed for this study. The study began with three patients. Treatment of subsequent participants was initiated after confirmation that no major safety issues were identified following completion of Cycle 1 by these three patients. Additional patients were added to obtain samples for pharmacokinetic analysis from 12 patients who completed Cycle 1 at the fixed, full starting dose.

As this was a noncomparative study, results are summarized in a descriptive manner according to the type and nature of the data (i.e., frequency and percentage for categorical variables; median and range or 95 % confidence intervals [Kaplan–Meier estimates] for continuous variables, and geometric mean and % coefficient of variance for pharmacokinetic parameters).

Data were analyzed by Bayer Yakuhin, Ltd., using SAS software (SAS Institute Inc., Cary, NC, USA).

## Results

The results presented here are from the final data analysis following the data cutoff on April 5th, 2011.

### Patient characteristics

Sixteen patients were enrolled into this study between July 2009 and April 2010. One patient was not treated because of screening failure, and the remaining 15 patients received study treatment. The median age was 59 years, with a range of 34–68 years. The baseline characteristics for all patients are presented in Table 1. All patients had an ECOG performance status of 0 or 1. The most common tumor site was the pancreas (six patients with ductal adenocarcinoma and one with neuroendocrine tumor). Thirteen patients had been previously treated with chemotherapy, 12 had previously undergone surgery, and three had received radiotherapy. One patient had previously received four lines of chemotherapy, one patient had received three lines, six patients had received two lines, and five patients had received one line. Two patients had never received chemotherapy.

**Table 1:** Patient demographics and baseline characteristics

		Regorafenib 160 mg starting dose
		(*N* = 15)
		*n* (%)
Sex	Male	11 (73)
	Female	4 (27)
European Cooperative Oncology Group performance status	0	12 (80)
	1	3 (20)
Tumor site	Pancreatic ductal adenocarcinoma	6 (40)
	Pancreatic neuroendocrine tumor	1 (7)
	Carcinoid tumor	2 (13)
	Gastric cancer (adenocarcinoma)	1 (7)
	Small-intestine carcinoma	1 (7)
	Penile carcinoma	1 (7)
	Cholangiocarcinoma	1 (7)
	Submandibular-gland neoplasm	1 (7)
	Urothelial tumor	1 (7)
Previous anticancer therapy	Systemic therapy^a^	13 (87)
	Surgery	12 (80)
	Radiotherapy	3 (20)
Number of previous chemotherapy regimens	0	2 (13)
	1	5 (33)
	2	6 (40)
	3	1 (7)
	4	1 (7)

^a^Systemic therapy included S-1 (*n* = 9), gemcitabine (*n* = 9), platinum compounds (*n* = 5), 5-fluorouracil (*n* = 3), taxanes (*n* = 2), and irinotecan (*n* = 1); patients may have had more than one type of chemotherapy either in combination or as separate lines of treatment

These 15 patients were included in the safety, pharmacokinetics, and efficacy analyses.

### Exposure to study medication

At the cutoff date of April 5th, 2011, one patient was still on treatment at Cycle 21. The median number of cycles received was 2 (range, 1–21 cycles). Duration of treatment (including off days) ranged from 0.9 to 20.1 months, and the median duration was 2.1 months.

No patient experienced any unacceptable toxicity in Cycle 1 according to the predefined criteria. Twelve patients (80 %) received the dose as planned in Cycle 1. Six patients required dose reduction (including two patients in Cycle 1), seven patients required interruption of regorafenib during daily treatment, and four patients delayed the start of their next cycle for longer than 7 days; in all cases the reason was adverse events. The most common reason for discontinuation was disease progression (*n* = 12), followed by adverse events (liver enzyme elevation *n* = 1; anemia *n* = 1).

### Safety

All 15 patients experienced at least one drug-related treatment-emergent adverse event of any grade (Table 2). Drug-related adverse events leading to dose interruption or dose reduction occurred in eight patients (Table 3). The most frequent adverse events were gastrointestinal (87 %), dermatologic (73 %), and hematologic (67 %). The most common gastrointestinal adverse event was diarrhea (67 %); one patient experienced grade 3 diarrhea. The most common dermatologic adverse event was hand–foot skin reaction (67 %), and this toxicity could be generally managed using local therapies, although two patients required dose reduction. The most common hematologic adverse events were anemia (40 %), lymphopenia (33 %), leukopenia (33 %), and thrombocytopenia (27 %); five patients experienced grade 3 lymphopenia, one patient had grade 3 anemia, another had grade 3 leukopenia, and no grade 3 thrombocytopenia was reported.

**Table 2:** Incidence of drug-related treatment-emergent adverse events and biochemical toxicities occurring at any point during the study

	Any grade	Grade 3 or 4
	*n* (%)	*n* (%)
Hematologic adverse events		
Anemia	6 (40)	1 (7)
Lymphopenia	5 (33)	4 (27)
Leukopenia	4 (27)	1 (7)
Thrombocytopenia	4 (27)	0
Nonhematologic adverse events		
Hand–foot skin reaction	10 (67)	2 (13)
Diarrhea	10 (67)	1 (7)
Hypophosphatemia	8 (53)	4 (27)
AST elevation	8 (53)	2 (13)
ALT elevation	7 (47)	2 (13)
Proteinuria	7 (47)	1 (7)
Hypoalbuminemia	7 (47)	0
Lactate dehydrogenase elevation	7 (47)	0
Alopecia	6 (40)	0
Weight loss	6 (40)	0
Anorexia	5 (33)	0
Constipation	5 (33)	0
Fatigue	5 (33)	0
Hypertension	5 (33)	0
Voice changes	5 (33)	0
Rash/desquamation	4 (27)	0
Biochemical toxicities^a^		
ALP elevation	14 (93)	2 (13)
Hypoalbuminemia	14 (93)	0
AST elevation	13 (87)	3 (20)
ALT elevation	12 (80)	3 (20)
Hypophosphatemia	12 (80)	4 (27)
Proteinuria	12 (8)	1 (7)
Hypocalcemia	9 (60)	0

*AST* aspartate aminotransferase, *ALT* alanine aminotransferase, *ALP* alkaline phosphatase

^a^Biochemical toxicities that occurred at any time after the start of study treatment, including those not reported as adverse events or being related to study drug

**Table 3:** Details of drug-related treatment-emergent adverse events resulting in treatment reduction or interruption

Patient	Primary tumor	Adverse event	Cycle	Dose change	Grade	Outcome
1	Pancreatic cancer	Leukopenia	1	Interrupted	2	Resolved
		Thrombocytopenia	1	Interrupted	2	Resolved
		AST elevation	1	Interrupted	3	–
		ALT elevation	1	Interrupted	3	–
		AST elevation	2	Reduced	4	Unchanged
		ALT elevation	2	Reduced	4	Unchanged
2	Carcinoid tumor	Diarrhea	3	Reduced	3	Resolved
		Hand–foot skin reaction	7	Interrupted	3	Unchanged
3	Submandibular gland neoplasm	Dental pulpitis	3	Interrupted	2	Resolved
4	Pancreatic cancer	Hand–foot skin reaction	3	Reduced	1	–
			9	Reduced	1	Resolved
		Back pain	11	Interrupted	3	Resolved
		Back pain	12	Reduced	–	–
5	Pancreatic cancer	Hypertension	1	Interrupted and reduced	2	Resolved
		Hand–foot skin reaction	2	Interrupted	3	–
			3	Reduced	1	Resolved
6	Carcinoid tumor	Proteinuria	9	Interrupted	3	–
		Proteinuria	10	Reduced	2	Resolved
		Gingivitis	18	Interrupted	2	Resolved
7	Gastric cancer	AST elevation	2	Interrupted	3	Resolved
8	Small-intestine carcinoma	Neutropenia	1	Interrupted and reduced	3	Resolved

*AST* aspartate aminotransferase, *ALT* alanine aminotransferase

The most common biochemical abnormalities occurring at any time after start of study treatment, whether or not they were reported as an adverse event or related to study drug, were alkaline phosphatase (ALP) elevation and hypoalbuminemia (93 % each), AST elevation (87 %), and ALT elevation, hypophosphatemia, and proteinuria (80 % each). Grade 3 or higher ALP elevation, AST or ALT elevation, hypophosphatemia, and proteinuria were reported in two, three, four, and one patients, respectively.

Serious adverse events were reported in six patients in total and were deemed to be treatment-related in three patients: one patient with pancreatic ductal cancer had grade 3 AST and ALT elevations before Cycle 2; one patient with gastric cancer developed grade 3 AST and ALT elevations and grade 2 disseminated intravascular coagulation—although AST and ALT levels improved immediately after dose interruption, the coagulation disorder remained unchanged at the final visit; and one patient with a pancreatic neuroendocrine tumor developed grade 3 polymyalgia rheumatica in Cycle 11, which resolved with pain management and regorafenib dose reduction in Cycle 12. No treatment-related deaths were recorded.

No clear changes were observed in vital signs or electrocardiogram findings during or after the study.

### Pharmacokinetics

Phamacokinetic results after the initial single dose (Cycle 0, Day 1) were obtained from all 15 patients. Thereafter, 12 patients were eligible for pharmacokinetic evaluation after multiple dosing (Cycle 1, Day 21). Three patients were excluded because of dose interruption or dose reduction during Cycle 1.

**Fig. 1:**
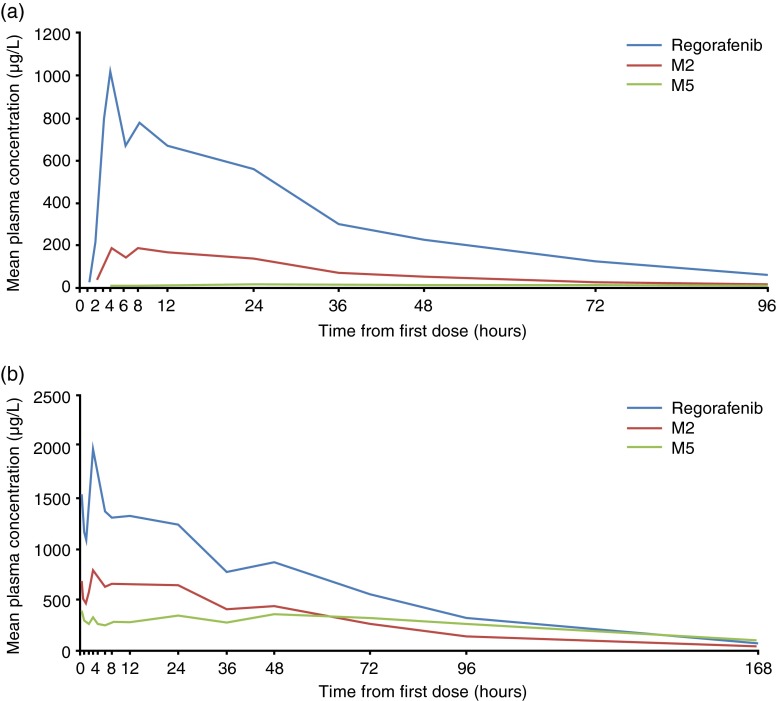
Plasma concentration profiles of regorafenib and its metabolites M2 (*N*-oxide metabolite) and M5 (*N*-oxide/*N*-desmethyl metabolite). **a** After a single oral dose of regorafenib 160 mg in Cycle 0. **b** After 21 days of dosing with regorafenib 160 mg once daily during Cycle 1. Data are geometric means, calculated from before the regorafenib dose during single dosing in Cycle 0 (time 0; *n* = 15) and before the regorafenib dose on day 21 of Cycle 1 (time 0; *n* = 11 for the pre-dose measurement, *n* = 12 for all other timepoints)

Table 4 shows the pharmacokinetic results after single dosing (Cycle 0, Day 21) and once-daily dosing for 21 days (Cycle 1, Day 21) for regorafenib and its metabolites M2 and M5. Figure 1 shows the geometric mean plasma concentration profiles of regorafenib and its metabolites after single and multiple dosing.

**Table 4:** Pharmacokinetics of regorafenib and its metabolites after single dosing (Cycle 0 Day 1) and multiple dosing (Cycle 1 Day 21)

	Regorafenib	M2	M5
Cycle 0, day 1	*N* = 15		
* C*_max_ (μg/L)	1,373.6 (108.0)	273.3 (388.9)	31.1 (167.3)^a^
AUC (μg.h/L)	34,559.8 (84.2)	7,820.6 (301.2)	3,441.8 (112.0)^a^
AUC_0–24_ (μg.h/L)	16,357.6 (86.1)	3,697.2 (340.5)	380.5/164.2 (112.0)^a^
* t*_1/2_ (h)	27.4 (29.9)	24.8 (27.7)	60.8 (78.2)^a^
* t*_max_ (h)	4.0 (1.9–8.1)	4.3 (2.8–24.0)	24.0 (2.8–71.3)^a^
Cycle 1, day 21	*N* = 12		
* C*_max_ (μg/L)	2,522.2 (77.0)	1,040.3 (213.7)	515.1 (413.9)
AUC_0–24_ (μg.h/L)	33,042.8 (68.5)	15,623.0 (212.5)	7,118.4 (458.6)
* t*_1/2_ (h)	30.4 (26.2)	29.5 (24.1)	57.5 (33.7)
* t*_max_ (h)	3.6 (0.6–47.9)	4.3 (0.6–47.9)	35.6 (0.6–73.2)
Accumulation ratio			
R_A_AUC	2.1 (64.6)	5.2 (136.9)	37.3 (109.3)^b^
R_A_ *C*_max_	2.0 (78.3)	4.8 (131.5)	36.0 (80.5)^b^

Data are geometric means (% coefficient of variance), except for *t*_max_, where data are median (range)

*AUC* area under the concentration–time curve; *AUC*_*0–24*_ AUC from 0 to 24 h; *C*_*max*_ maximum concentration; *M2* regorafenib *N*-oxide metabolite; *M5* regorafenib *N*-oxide/*N*-desmethyl metabolite; *R*_*A*_ *AUC* accumulation ratio calculated from the AUC_T_ (expected dosing interval) obtained after multiple dosing and AUC_T_ after single dosing; *R*_*A*_ *C*_*max*_ accumulation ratio calculated from *C*_max_ after multiple dosing and *C*_max_ after single dosing; *t*_*1/2*_ terminal half life; *t*_*max*_ time to maximum concentration

^a^ *n* = 13

^b^ *n* = 10

After single dosing, the median time to maximum concentration (*t*_max_) of regorafenib and M2 was 4 h, and that of M5 was 24 h. Similar terminal half lives (*t*_1/2_) were observed for regorafenib and M2, being approximately 27 h and 25 h (geometric means), respectively, and a longer *t*_1/2_ of approximately 61 h was observed for M5.

There was no significant change in *t*_max_ and *t*_1/2_ after single dosing (Cycle 0, Day 1) and multiple dosing (Cycle 1, Day 21) for each analyte. After once-daily multiple dosing for 21 days, the area under the concentration–time curve (AUC) from 0 to 24 h was 2.1-fold higher for regorafenib, 5.2-fold higher for M2, and 37.3-fold higher for M5 than after the single dose (see R_A_AUC in Table 4). After continuous dosing, the maximum concentration was 2.0-fold, 4.8-fold, and 36.0-fold higher, respectively, than after the single dose (see R_A_ *C*_max_ in Table 4). The AUC from 0 to 24 h of M2 accounted for 47.3 % of regorafenib on Day 21 in Cycle 1. That of M5 accounted for 21.5 %.

Two out of the six patients who experienced serious adverse events showed lower plasma concentration levels than were generally seen in the overall study population on Day 21 in Cycle 1. The lower absorption observed in these patients may be due to poor solubilization of regorafenib caused by impaired bile excretion. The other four patients who experienced serious adverse events showed no differences in pharmacokinetics compared with the study population.

### Efficacy

Partial response was observed in one patient (who had a pancreatic neuroendocrine tumor), with a duration of response of 10.5 months; no patient achieved a complete response. Thus the overall response rate was 7 % (95 % confidence interval 0.2–31.9 %). Seven patients had stable disease (four with pancreatic ductal adenocarcinoma, two with carcinoid tumor, and one with submandibular gland neoplasm), one of whom was still receiving regorafenib (in Cycle 21) at the data cutoff. The median time to progression was 3.7 months (95 % confidence interval 1.9–12.4 months).

## Discussion

In this study, conducted to evaluate safety, pharmacokinetics, and preliminary efficacy in Japanese patients with treatment-refractory solid tumors, the broad-spectrum receptor kinase inhibitor regorafenib showed acceptable tolerability and antitumor activity.

The dose and schedule of regorafenib treatment was selected in a phase I dose-escalation study involving European patients who received regorafenib doses from 10 to 220 mg per day in a discontinuous schedule (21 days on treatment, 7 days off treatment) [5]. Signs of antitumor activity were observed at doses from 60 to 220 mg in that study [5], and efficacy was confirmed at 160 mg/day in a phase II study involving patients with renal cell carcinoma [7]. In vitro investigations showed that regorafenib is metabolized by cytochrome p450 3A4 and UDP-glucuronosyltransferase 1A9 glucuronidation [11]. As there is no evidence suggesting ethnic differences in polymorphism for these enzyme pathways, the possibility of ethnic differences in metabolism was considered to be low, and drug exposure in Japanese patients was not expected to differ markedly from that in European patients. Therefore, a fixed starting dose of 160 mg once daily was chosen for this study to evaluate feasibility in the Japanese population before participation in the global phase III studies in metastatic CRC and GIST.

The most common drug-related toxicities, including hand–foot skin reaction, diarrhea, hypophosphatemia, and liver transaminase elevation, were mostly mild or moderate and improved after dose interruption. Dermatologic adverse events such as hand–foot skin reaction and rash were frequently observed. Hand–foot skin reaction was reported in ten patients, including two who experienced this toxicity at grade 3. Local therapies and prophylaxis, such as moisturizing cream, were useful to manage this dermatologic toxicity. Gastrointestinal adverse events such as diarrhea and anorexia were also frequently observed. Diarrhea was reported in ten patients, including one patient who experienced grade 3 diarrhea. This adverse event was mostly mild or moderate and easily managed. A single case of grade 3 polymyalgia rheumatica was considered to be related to study drug. Pain associated with this condition was managed pharmacologically, and symptoms had resolved by the end of the study.

Table 5 shows a comparison of data, including the most frequent adverse events, from Japanese and European populations in clinical studies of regorafenib. The frequency of hand–foot skin reaction was 67 % in our study and 67–71 % in the European phase I and II studies [5–8], indicating similarity between Japanese and European populations. Although the patient population was small in each study, resulting in some variation between studies, the frequency of other adverse events, such as diarrhea, voice changes, hypertension, fatigue, and anorexia, seemed to be consistent between studies. In this study of Japanese patients, the frequency of liver enzyme elevations as a drug-related adverse event was higher than in studies in European populations [5–10]. The incidence of biochemical toxicities (overall and grade ≥3) occurring at any time after the start of study treatment, whether or not they were reported as an adverse event or related to study drug, seemed to be similar between our study and the 160 mg dose cohort in the European phase I study [5]. Comparing our Japanese phase I study with the European phase II study showed some differences in incidence, which might reflect differences in the target populations: the phase II study enrolled patients receiving first-line treatment for renal cell carcinoma [7], whereas our Japanese study enrolled patients with heavily pretreated solid tumors. It is, however, important to note that this was a small group of Japanese patients, and these comparative differences or similarities have not yet been confirmed in larger studies.

**Table 5:** Comparison of Japanese and European populations from clinical studies of regorafenib

	Japan phase I study	European phase I study [5]	European phase II study [7]
Patients (*n*)	15	12^a^	49
Age (years)			
Median (range)	59 (34–68)	60 (20–77)^b^	62 (40–76)
European Cooperative Oncology Group performance status	0–1	0–2^b^	0–1
Disease status	Advanced solid tumors	Advanced solid tumors	Advanced renal cell carcinoma
Treatment history	0–4 previous systemic treatments	Previously treated	Untreated
Adverse events (%)			
All grades (grade 3/4)			
Hand–foot skin reaction	67 (13)	67 (25)	71 (33)
Diarrhea	67 (7)	50 (17)	45 (10)
Voice changes/hoarseness	33 (0)	42 (0)	35 (0)
Hypertension	33 (0)	50 (17)	49 (6)
Fatigue	33 (0)	17 (0)	53 (8)
Alopecia	40 (0)	33 (0)	45 (0)
Rash	27 (0)	50 (8)	39 (6)
Anorexia	33 (0)	50 (8)	29 (6)
Incidence of biochemical toxicities occurring at any time after start of study treatment, whether or not they were reported as an adverse event or related to study drug (%)			
All grades (grade 3/4)			
AST	87 (20)	75 (25)	41 (2)
ALT	87 (20)	75 (25)	39 (2)

*AST* aspartate aminotransferase, *ALT* alanine aminotransferase

^a^160 mg cohort only

^b^Includes 53 patients in all dose cohorts

The pharmacokinetic profiles of regorafenib, M2, and M5 demonstrated lower mean exposure in the Japanese patients than in the European population [5]. However, large inter-individual variability was observed in this study, and the range of regorafenib exposure was similar to those reported in European patients in phase I and II studies [5–7]. In vitro analysis of regorafenib and its metabolites, including biochemical enzyme assays, a competitive kinase binding assay, and cellular binding assays, has previously demonstrated M2 and M5 to be pharmacologically active metabolites of regorafenib (data not shown). However, considering protein-binding properties and the concentrations of unbound regorafenib and its metabolites, regorafenib provides the major factor in pharmacological activity at steady state. Further analysis in larger cohorts of patients would help to confirm the likely exposure to regorafenib in different ethnic populations and at different tumor sites.

More than half of the participants in our study achieved tumor control with regorafenib (partial response *n* = 1; stable disease *n* = 7), indicating that regorafenib has a clinical benefit and warrants further clinical research in Japanese patients. Of particular interest was the partial response in a patient with pancreatic neuroendocrine tumor; currently, there are few treatment options for patients with such tumors, and exploring the potential role of regorafenib in this setting could be of value.

This study provides evidence that regorafenib can be given to Japanese patients without undue toxicity and indicates that antitumor efficacy can be expected. The regorafenib dosing schedule in international phase III trials (160 mg orally once daily, 21 days on/7 days off treatment) was therefore considered appropriate for the Japanese patient population.
